# Robotics in Breast Surgery: Current Advantages, Disadvantages, and Applications

**DOI:** 10.7759/cureus.87972

**Published:** 2025-07-15

**Authors:** Tiago Branco, Ana Rodrigues, Ana Rita Martins

**Affiliations:** 1 General Surgery, Unidade Local de Saúde do Oeste, Hospital de Caldas da Rainha, Caldas da Rainha, PRT; 2 General Surgery, Unidade Local de Saúde de Lisboa Ocidental, Hospital de São Francisco Xavier, Lisboa, PRT

**Keywords:** breast cancer, breast surgery, innovation surgery, robotic breast surgery, robotic surgery

## Abstract

Breast cancer represents the most prevalent malignancy among women, requiring surgical procedures including mastectomies and lumpectomies. While conventional surgical procedures are typically more invasive, robot-assisted breast surgery has emerged as a minimally invasive alternative to traditional mastectomy and reconstruction techniques. Robotic systems offer enhanced precision, three-dimensional (3D) visualization, and improved ergonomics. Their primary objectives are to minimize invasiveness, optimize the surgeon's visibility, reduce postoperative complications, and achieve superior aesthetic outcomes. Robotic nipple-sparing mastectomy demonstrates oncological safety comparable to conventional methods, with lower overall complication rates.

However, significant challenges persist, including high initial costs, longer operative times, and the imperative for specialized training. While robotic surgery enhances surgeon ergonomics and mitigates fatigue, further formal clinical studies are required to conclusively validate these benefits. Current applications encompass nipple-sparing mastectomies, sentinel lymph node biopsies, and breast reconstruction procedures.

The document also explores robotic techniques for breast reconstruction, highlighting potential benefits such as reduced postoperative pain and improved aesthetic satisfaction. Despite promising preliminary results, more long-term oncological safety data and comprehensive cost-effectiveness analyses are essential for a complete evaluation of robotics' role in breast surgery. This review aims to summarize the current evidence on robotic-assisted breast surgery, focusing on its advantages, limitations, and clinical applications.

## Introduction and background

Based on GLOBOCAN 2022 statistics, breast cancer stands as the most frequently diagnosed malignant neoplasm in women and ranks second across both sexes. It is also the leading cause of cancer-related mortality in women and ranks fourth among all cancer deaths globally [1]. The disease exhibits marked aggressive characteristics, as malignant cells demonstrate considerable capacity for invasion and dissemination to essential organs like the brain and liver via circulatory and lymphatic systems, significantly threatening both patient survival and quality of life [2].

Surgical management of breast conditions includes diverse procedures designed to treat malignancies and manage benign pathologies. These procedures may also be performed to fulfill cosmetic objectives. Oncological breast patients typically undergo procedures including mastectomy or breast-conserving surgery to remove malignant or involved tissue. Surgical intervention coupled with reconstruction techniques represents vital therapeutic modalities for breast cancer management and patient recovery, while also addressing aesthetic enhancement requests [3,4]. Following initial surgery, reconstructive procedures can facilitate the restoration of anatomical form and psychological well-being. Furthermore, breast surgical interventions encompass aesthetic operations that enable patients to alter breast dimensions and contour [2,3].

In recent years, considerable advancements have occurred in breast surgery and reconstruction, focusing on improving technical accuracy, optimizing clinical results, and focusing on patients' mental and emotional well-being. These innovations have prompted considerable advancement in the discipline, especially through the integration of robotic surgery, which represents a major breakthrough in minimally invasive breast surgery [2-4]. Robot-assisted surgery has improved surgical technique, which was limited by the capabilities of the human body, thanks to diverse advantages. These advantages include increasing the degree of freedom of movement, eliminating tremors, providing expanded three-dimensional (3D) vision, achieving ergonomic positioning, and offering better resolution. These improvements yield reduced complication rates, shortened rehabilitation periods, and refined aesthetic achievements. Moreover, the adoption of robotic assistance for breast operations enables less invasive approaches, thereby reducing tissue trauma and enhancing patient comfort. Over time, continuous advancements in surgical techniques and technologies have significantly improved their safety and efficacy [2-4]. This has been particularly impactful in procedures such as skin and nipple-sparing mastectomy, which present challenges even for experienced surgeons. While robotic skin and nipple-sparing mastectomy offers improved access and ergonomics, important issues related to safety, oncological outcomes, and cost-effectiveness need to be addressed.

## Review

Objectives and methods

This manuscript focuses on analyzing the notable revolution that robotic applications have produced in breast surgical and reconstructive methodologies. The objectives are to offer an overview of the robotic technology available in breast surgery, describing the advantages and disadvantages of integrating it into clinical practice. Our methodology incorporated a structured evaluation of relevant studies examining robotic technology deployment in breast surgical procedures. Literature searches were conducted in PubMed between March and June 2025 including articles from 2016 to 2025, utilizing keywords like "breast surgery," "minimally invasive breast," "robotic breast surgery," "breast reconstruction," and "robotic surgery." Articles published in English were selected based on their contribution to understanding how current robotic technology influences surgical techniques, patient outcomes, and the associated advantages and disadvantages.

Types of robotic systems in breast surgery

There are several robotic surgical systems employed in breast surgery [3]. The da Vinci^®^ Surgical System (Intuitive Surgical, Inc., Sunnyvale, CA, US) remains a leading and extensively implemented system within robotic surgery, and this system is characterized by its extraordinary dexterity, precision, and control. Mazor^®^ X Stealth Edition (Medtronic Inc., Dublin, Ireland), initially conceived for spine surgeries, has been adapted for breast surgery. It stands out for its ability to integrate preoperative planning with intraoperative navigation [3]. The Senhance^®^ Surgical System (Asensus Surgical, Durham, NC, US) is characterized by distinctive attributes, including tactile response and eye-movement detection. Haptic feedback allows the surgical team to manipulate robotic instruments more intuitively, thus increasing precision and tactile sensation [3]. Versius^®^ (CMR Surgical, Cambridge, UK) benefits from its compact design and portability, ideal when the workspace is limited [3]. In recent times, other robotic surgical systems have been developed, such as the MUSA^®^ Surgical System (Microsure B.V., Son, The Netherlands) and the Symani^®^ Surgical System (Medical Microinstruments, Jacksonville, FL, US), affording greater operative versatility due to their high degree of freedom in wrist tools, which is especially beneficial for microsurgery [5].

Advantages

Perioperative Safety

In relation to perioperative safety, the studies by Toesca et al. evaluated the prevalence of nipple-areolar complex or skin perfusion issues, nipple viability loss, tactile impairment, seroma occurrence, hematoma formation, secondary surgical interventions, and infectious events. No significant differences were found in the number or type of complications between patients undergoing nipple-sparing mastectomy using robotic technique and conventional nipple-sparing mastectomy [6,7]. A comprehensive review with meta-analysis by De la Cruz-Ku et al. released in 2023 revealed that the rate of general complications was lower in the nipple-sparing mastectomy using robotic technique group than in the conventional nipple-sparing mastectomy group (RR = 0.68; 95% CI: 0.49-0.96) [8]. In a systematic review and meta-analysis, Nessa et al. observed a significant reduction in nipple necrosis rates after nipple-sparing mastectomy using the robotic technique [9].

Oncological Safety

Regarding oncological safety, recent data suggest that nipple-sparing mastectomy using robotic technique offers oncological safety comparable to conventional nipple-sparing mastectomy with respect to involved margins, loco-regional recurrence, disease-free survival, and overall survival [4]. A meta-analysis developed by De la Cruz-Ku et al. featured in the 2023 Journal of Robotic Surgery indicated equivalent rates of margin positivity between both methods, with no early recurrences after robotic skin and nipple-sparing mastectomy during a follow-up period ranging from 90 days to 32.1 months [8]. A single-center retrospective study conducted at Southwest Hospital in China and published in JAMA in 2022 analyzed approximately 2,400 patients who underwent minimally invasive breast surgery, including robotic, endoscopic, and endoscope-assisted techniques. This study concluded that there are no significant differences in five-, 10-, and 15-year survival rates when compared to conventional breast surgery [10].

Improved Technical Precision, Visualization, and Control

Robotic systems enable improved precision, visualization, and control with less visible scars, offering precise and delicate movements that are particularly helpful during tissue dissection, surgical stitching, and tumor resection, where minor deviations can result in major complications. These advantages lead to improved aesthetic results and enhanced patient satisfaction [4]. They offer eye-tracking capabilities that improve control, identification of blood vessels and nerves, support delicate maneuvers, and ensure precise delineation of tumor margins necessary in breast surgeries [4].

Ergonomics and Surgeon Well-Being 

The implementation of robotic systems represents an effective solution to mitigate physical effort and alleviate surgical exhaustion. These platforms convert the surgeon's hand motions into accurate robotic movements, decreasing the effort needed for instrument control. The improvement in ergonomics, facilitated by robotics, allows surgeons to experience less tension and physical exhaustion, ensuring optimal performance, especially during prolonged surgeries [3,5,11]. The adaptability offered by the robotic technique in confined anatomical regions helps maintain surgical precision, efficacy, better cosmetic results, and patient satisfaction [5]. In a comparative study carried out by Lai et al., the aesthetic efficacy of nipple-sparing mastectomy using robotic technique versus conventional nipple-sparing mastectomy was analyzed, in which 92% of patients undergoing nipple-sparing mastectomy using robotic technique rated their aesthetic result as "excellent" or "satisfactory," while only 75.6% of patients who underwent conventional nipple-sparing mastectomy reported the same level of satisfaction, with a statistically significant difference (P = 0.046) [12].

Disadvantages

Economic Constraints

In terms of disadvantages, high costs are an important issue and require considerable initial investment, mainly in the acquisition and maintenance of the necessary equipment, representing an economic challenge that can restrict its widespread use, especially in resource-limited environments. Given the high initial cost of equipment and its maintenance, concerns arise as to whether patients from different institutions, with varying levels of resources, will be able to equitably benefit from the advantages offered by robot-assisted surgery, especially in the field of breast surgery [4]. In a study carried out by Lai et al., cited by Doll et al., the economic aspect of nipple-sparing mastectomy using robotic technique and conventional nipple-sparing mastectomy was meticulously analyzed. It was observed that the average cost of robotic mastectomy was significantly higher, reaching $10,877 compared to $5,702 for conventional conservative mastectomy. This difference highlights the considerable financial investment associated with the use of robotic technology in surgical procedures. However, to date, there is insufficient information on long-term cost comparisons between both modalities [4].

Prolonged Operative Times

A factor that hinders the general adoption of robotic techniques is that it entails a significant increase in surgical time, compared to the conventional method, in addition to the limitations of robotic systems. A randomized study featured in the Annals of Surgery by Toesca et al. observed that the average operative length for unilateral nipple-conserving mastectomy through robotic technique was one hour and 18 minutes longer than when the conventional method was used [7]. Although most studies indicate that operative times are considerably longer with nipple-sparing mastectomy using robotic technique compared to conventional nipple-sparing mastectomy, some have suggested that this difference could be due to the learning curve [5]. A retrospective study published in the Annals of Surgical Oncology in 2019 showed that docking time was reduced from 20 to seven minutes, and the total duration of the unilateral robotic mastectomy procedure decreased to less than 100 minutes once competence was achieved [13].

Learning Curve and Training Limitations

Surgeons require exhaustive training to master robotic systems effectively, which can involve a steep learning curve and extensive practice to achieve optimal competence and ensure patient safety. Simulation and mentorship are fundamental to achieving competence in handling the robotic platform before treating real patients, as this allows professionals to increase their confidence and master the technology; furthermore, supervised cases are often a requirement for accreditation, supporting the formal certification process for surgeons [14,15]. Lai et al. state that approximately 13 procedures are necessary to achieve a notable decrease in operation duration, which highlights the crucial impact of the surgeon's experience as a determining factor [16].

Limited Haptic Feedback and Technical Challenges

The lack or limitation of touch sensation in robotic-assisted techniques may induce difficulties, as it reduces the surgeon's capability to evaluate tissue characteristics instantaneously. Despite having visual and auditory information, the lack of haptic sensation can lead to a sense of losing the distinctive surgeon’s tactile perception, which could negatively affect clinical judgment and the accuracy of specific surgical steps [4]. Robotic systems, like any other technology, can experience technical and software problems. These unforeseen setbacks may affect surgery, making it essential to have a rapid problem-solving capability. The eventuality of facing technical failures underscores the need for a highly experienced support team and standardized protocol procedures to manage these situations in the surgery theater. Such problems can add a degree of uncertainty and generate stress in the surgical environment, so it is essential that the surgical team and staff are ready to handle these difficulties proficiently, thereby securing patient protection and operative success [4].

Robotic applications in breast surgery

Simulation and 3D Modeling

There are several robotic applications in breast surgery. Surgical simulation allows for refining the medical strategy, familiarizing the staff with robotic control systems, and shortening the skill acquisition time in complex surgical procedures [4]. The acquisition of images and 3D modeling through robotic systems combined with cutting-edge imaging technologies, such as magnetic resonance imaging and computed tomography, to generate precise 3D models of the patient's breast is another use of robotics. Li and Li refer to an innovative technology developed by Duarte et al. able to produce depth maps and images, subsequently transformed in real time into 3D meshes for 3D breast reconstruction, given that this technology could predict breast dimensions and volume to determine prosthetic or tissue needs in reconstructive surgery, including 3D printing applications [5].

Sentinel Lymph Node Biopsy

In sentinel lymph node biopsy, detailed visualization of anatomy and the use of navigation tools in the operating room are essential both for identifying and completely excising tumor tissue during robotic breast surgery and for precisely detecting and resecting affected axillary lymph nodes. Robotic systems allow for precise localization and accurate excision of sentinel nodes, minimizing intra- and postsurgical complications (e.g., arm lymphedema) [4,5].

Robotic Nipple-Sparing Mastectomy

One of the main robotic applications is nipple-sparing mastectomy, which is capable of significantly improving aesthetic appearance without compromising oncological efficacy. This minimally invasive strategy, with improved cosmetic results for nipple preservation surgery, may enhance patients' overall life quality. A meta-analysis reveals that, compared to traditional nipple-sparing mastectomy, the robotic version shows no significant differences in postoperative complications; it presents as a safe option with greater aesthetic satisfaction compared to the conventional method [17]. In a study carried out by Sanson et al., 79 patients who underwent 138 nipple-sparing mastectomies using robotic technique procedures, complemented by immediate prosthetic reconstruction, were analyzed [18]. The aesthetic results were generally very satisfactory, the quality of life of the patients remained without significant changes after the intervention, and it was associated with low rates of major necrosis, being a safe and replicable technique enabling scar-free breast reconstruction [18].

Breast Reconstruction With Latissimus Dorsi Flap

Robotic latissimus dorsi reconstruction delivers benefits of shorter incisions and overcomes endoscopic procedural limitations. Fouarge and Cuylits demonstrated that robot-assisted latissimus dorsi flap collection is a safe, replicable, and efficient procedure, enabling precise surgical control with minimal thoracic scar [19]. Eo et al., in a prospective study conducted at a single institution, compared the conventional, endoscopic, and robotic methods and found no statistically significant difference between the three methods regarding postoperative opioid analgesic dosage, length of hospital stay, or total average drainage amount at the donor site during hospitalization. As for patient satisfaction after surgery, especially in relation to the scar at the donor site, the conventional method yielded a significantly lower score than the endoscopic and robotic techniques [20].

*Deep Inferior Epigastric Perforator Flap* 

Nowadays, the deep inferior epigastric perforator (DIEP) has emerged as the primary option for autologous breast reconstruction. However, despite technological advancements, obtaining the DIEP flap using standard open methods still requires a substantial incision. Because the DIEP pedicle is located in the deep layer of the rectus abdominis muscle, its dissection commonly involves cutting this muscle, which inflicts direct damage. This can affect the motor nerves of the rectus abdominis, creating muscle dysfunction and important postsurgical pain [5]. In a retrospective study, 207 patients who underwent mastectomy with breast reconstruction using a DIEP flap, using either a conventional or robotic approach, between July 2017 and January 2021, were analyzed. It was observed that the robotic method presented a significantly longer mean reconstruction time (P < 0.001) and patients experienced significantly less postoperative pain (P = 0.001) and a shorter hospital stay (P = 0.002) and obtained superior scores in the abdominal physical well-being domain according to BREAST-Q (P = 0.020) [21].

Incorporating artificial intelligence and augmented reality technologies, Mavioso et al. conducted a pilot study that employed machine learning algorithms to analyze vascular imaging from computed tomography scans in patients undergoing DIEP flap breast reconstruction. Their findings demonstrated that this technology has the potential to significantly reduce preoperative planning time [5,22].

Discussion

The integration of robotics in breast surgery represents a significant advancement, with the potential to improve outcomes in terms of surgical precision, safety, and patient satisfaction. However, the incorporation of these technologies is not without challenges and critical considerations.

One of the strengths of robotic surgery is its ability to provide enhanced 3D visualization and greater dexterity in instrument manipulation, which can result in more precise and less invasive procedures. This is particularly relevant in procedures such as skin-sparing and nipple-sparing mastectomies, where the preservation of aesthetics is a significant objective achieved through the use of a smaller incision located outside the mammary skin area. Studies suggest that robotic surgery may achieve superior aesthetic outcomes and greater patient satisfaction compared to conventional techniques [4,5,7,12].

Nevertheless, it is essential to address the limitations and challenges associated with robotic surgery. The high initial investment and ongoing maintenance costs of robotic systems can restrict their availability in many medical centers. This limited accessibility raises important questions about equity in access to advanced surgical technology. Additionally, prolonged operative times and the need for specialized training for surgeons are factors that must be considered when implementing robotic surgery [7,8,15].

Although initial studies suggest that robotic surgery is safe and effective from an oncological perspective, further long-term research is needed to confirm these findings and to evaluate the impact on survival and recurrence of breast cancer [10]. Furthermore, cost-effectiveness analyses are necessary to determine whether the clinical and aesthetic benefits of robotic surgery justify the additional investment.

A new release of the Consensus Statement on Robotic Nipple Sparing Mastectomy Expert Panel was released in June 2025. The expert panel agreed that nipple-sparing mastectomy using robotics demonstrates oncologic safety equivalent to traditional methods, with the added benefits of improved aesthetic outcomes and greater patient satisfaction. The consensus established appropriate candidates as those requiring prophylactic mastectomy, patients with early-stage disease (Tis-T2), and carefully selected T3 cases following neoadjuvant treatment, while excluding patients with inflammatory breast cancer, nipple involvement, or tumors within 2 cm of the nipple-areolar complex [23]. In the future, the integration of artificial intelligence and augmented reality in robotic surgery could open new possibilities for preoperative planning, intraoperative navigation, and treatment personalization.

## Conclusions

Robotic surgery in the field of breast surgery and reconstruction represents a promising evolution, offering significant advantages in terms of precision, visualization, and the potential to improve aesthetic outcomes and patient satisfaction. Robotic systems such as the da Vinci and others have proven to be valuable tools in procedures including skin- and nipple-sparing mastectomies, sentinel lymph node biopsy, and flap-based breast reconstruction. However, it is crucial to recognize and address the limitations and challenges associated with this technology. High costs, prolonged operative times, and the need for specialized training are factors that should be carefully considered when implementing robotic surgery programs, alongside the need for long-term studies to confirm oncological safety and assess the impact on survival and recurrence in breast cancer.

Despite these challenges, the potential for robotic surgery to transform breast cancer care is undeniable. With the continued advancement of technology and the integration of automation, artificial intelligence, and augmented reality, new applications and improved outcomes are likely to emerge in the future. However, it is essential that the adoption of these technologies is based on robust evidence and carried out ethically and responsibly, with patient well-being and safety always as the highest priorities.

## References

[1] Bray F, Laversanne M, Sung H, Ferlay J, Siegel RL, Soerjomataram I, Jemal A (2024). Global cancer statistics 2022: GLOBOCAN estimates of incidence and mortality worldwide for 36 cancers in 185 countries. CA Cancer J Clin.

[2] Park KU, Cha C, Pozzi G (2023). Robot-assisted nipple sparing mastectomy: recent advancements and ongoing controversies. Curr Breast Cancer Rep.

[3] Jain Y, Lanjewar R, Shinde RK (2024). Revolutionising breast surgery: a comprehensive review of robotic innovations in breast surgery and reconstruction. Cureus.

[4] Doll A, Kopkash K, Baker J (2024). Emerging role of robotic surgery in the breast. Clin Breast Cancer.

[5] Li T, Li C (2025). Emerging trends in robotic breast surgery in the era of artificial intelligence. Plast Aesthet Res.

[6] Toesca A, Peradze N, Manconi A (2017). Robotic nipple-sparing mastectomy for the treatment of breast cancer: feasibility and safety study. Breast.

[7] Toesca A, Sangalli C, Maisonneuve P (2022). A randomized trial of robotic mastectomy versus open surgery in women with breast cancer or BrCA mutation. Ann Surg.

[8] De la Cruz-Ku G, Chambergo-Michilot D, Perez A (2023). Outcomes of robotic nipple-sparing mastectomy versus conventional nipple-sparing mastectomy in women with breast cancer: a systematic review and meta-analysis. J Robot Surg.

[9] Nessa A, Shaikh S, Fuller M, Masannat YA, Kastora SL (2024). Postoperative complications and surgical outcomes of robotic versus conventional nipple-sparing mastectomy in breast cancer: meta-analysis. Br J Surg.

[10] Wan A, Liang Y, Chen L (2022). Association of long-term oncologic prognosis with minimal access breast surgery vs conventional breast surgery. JAMA Surg.

[11] Kopkash K, Novak K, Kuchta K (2019). The "nipple whipple"?! A pilot study to assess the ergonomic effects of nipple-sparing mastectomy. Ann Surg Oncol.

[12] Lai HW, Chen ST, Mok CW (2020). Robotic versus conventional nipple sparing mastectomy and immediate gel implant breast reconstruction in the management of breast cancer-a case control comparison study with analysis of clinical outcome, medical cost, and patient-reported cosmetic results. J Plast Reconstr Aesthet Surg.

[13] Lai HW, Chen ST, Lin SL (2019). Robotic nipple-sparing mastectomy and immediate breast reconstruction with gel implant: technique, preliminary results and patient-reported cosmetic outcome. Ann Surg Oncol.

[14] (2025). Royal Australasian College of Surgeons. RACS robot-assisted surgery courses. https://www.surgeons.org/Education/RACS-robot-assisted-surgery-courses#RoboSET%20Robotic%20Simulation%20skills%20course.

[15] Green CA, Levy JS, Martino MA, Porterfield JR Jr (2020). The current state of surgeon credentialing in the robotic era. Ann Laparosc Endosc Surg.

[16] Lai HW, Wang CC, Lai YC (2019). The learning curve of robotic nipple sparing mastectomy for breast cancer: an analysis of consecutive 39 procedures with cumulative sum plot. Eur J Surg Oncol.

[17] Filipe MD, de Bock E, Postma EL (2022). Robotic nipple-sparing mastectomy complication rate compared to traditional nipple-sparing mastectomy: a systematic review and meta-analysis. J Robot Surg.

[18] Sanson C, Roulot A, Honart JF, Rimareix F, Leymarie N, Sarfati B (2021). Robotic prophylactic nipple-sparing mastectomy with immediate prosthetic breast reconstruction: a prospective study of 138 procedures. Chirurgia (Bucur).

[19] Fouarge A, Cuylits N (2020). From open to robotic-assisted latissimus dorsi muscle flap harvest. Plast Reconstr Surg Glob Open.

[20] Eo PS, Kim H, Lee JS, Lee J, Park HY, Yang JD (2023). Robot-assisted latissimus dorsi flap harvest for partial breast reconstruction: comparison with endoscopic and conventional approaches. Aesthet Surg J.

[21] Lee MJ, Won J, Song SY (2022). Clinical outcomes following robotic versus conventional DIEP flap in breast reconstruction: a retrospective matched study. Front Oncol.

[22] Mavioso C, Araújo RJ, Oliveira HP (2020). Automatic detection of perforators for microsurgical reconstruction. Breast.

[23] Ryu JM, Mok CW, Toesca A (2025). Consensus statement on robotic nipple sparing mastectomy expert panel. J Breast Cancer.

